# Pre-Experimental Wet Heat Sterilization Alters the Ecotoxicity of Pristine Graphene Oxide Toward *Daphnia magna*

**DOI:** 10.3390/nano15231800

**Published:** 2025-11-28

**Authors:** Ildikó Fekete-Kertész, Péter Hajdinák, Krisztina László, Anna Bulátkó, Viktor Podhragyai, Benjámin Sándor Gyarmati, Zoltán Molnár, Mónika Molnár

**Affiliations:** 1 Environmental Microbiology and Biotechnology Group, Department of Applied Biotechnology and Food Science, Faculty of Chemical Technology and Biotechnology, Budapest University of Technology and Economics, Műegyetem rkp. 3., H-1111 Budapest, Hungary; molnar.monika@vbk.bme.hu; 2 Laboratory of Biochemistry and Molecular Biology, Department of Applied Biotechnology and Food Science, Faculty of Chemical Technology and Biotechnology, Budapest University of Technology and Economics, Műegyetem rkp. 3., H-1111 Budapest, Hungary; hajdinak.peter@vbk.bme.hu (P.H.); podhragyai.viktor@vbk.bme.hu (V.P.); 3 Surface Chemistry Group, Department of Physical Chemistry and Materials Science, Faculty of Chemical Technology and Biotechnology, Budapest University of Technology and Economics, Műegyetem rkp. 3., H-1111 Budapest, Hungary; laszlo.krisztina@vbk.bme.hu (K.L.); bulatko.anna@vbk.bme.hu (A.B.); 4 Soft Matters Group, Department of Physical Chemistry and Materials Science, Faculty of Chemical Technology and Biotechnology, Budapest University of Technology and Economics, Műegyetem rkp. 3., H-1111 Budapest, Hungary; gyarmati.benjamin@vbk.bme.hu; 5 Department of Plant Sciences, Faculty of Agricultural and Food Sciences, Széchenyi István University, Vár Sqr. 2., H-9200 Mosonmagyaróvár, Hungary; molnar.zoltan@sze.hu

**Keywords:** *Daphnia magna*, nanoecotoxicology, graphene oxide, oxidative stress, heartbeat rate, feeding activity

## Abstract

As the exposure of the aquatic ecosystem to graphene oxide (GO) increases with its growing production and use, understanding the structure–property–toxicity relationships becomes increasingly critical in the development of effective safe design guidelines. An appropriate testing methodology is crucial in ecotoxicity assessments to accurately characterize the environmentally relevant toxicity of nanoparticles, particularly for GO, where the physicochemical properties fundamentally determine their interactions and toxicity toward aquatic organisms. Many ecotoxicological methods require the heat sterilization of samples as a preliminary treatment prior to analysis. To investigate changes in toxicity profiles induced by wet heat sterilization pretreatments (autoclaving and Tyndall treatment) of a well-characterized GO product, a comprehensive ecotoxicological evaluation was performed with *Daphnia magna*. This included conventional lethality and immobilization tests, along with sublethal endpoints such as heart rate and feeding activity, supplemented with the analysis of oxidative stress biomarkers. Physicochemical alterations in GO due to sterilization were examined with dynamic light scattering, ultraviolet-visible, and thermogravimetry/mass spectrometry. Sublethal endpoints were shown to be more sensitive indicators of toxicity than conventional methods, with feeding activity and heart rate inhibition demonstrating time and concentration-dependent effects. Heat-sterilized GOs exhibited greater ecotoxicity compared to pristine GO, as evidenced by elevated ROS levels and increased oxidative stress biomarkers (GPx and GST activities), implicating oxidative stress as a central mechanism of toxicity. Despite the subtle differences observed in the physicochemical properties, the impact of heat sterilization on toxicity is clear. Our research underscores the critical importance of adopting appropriate testing and evaluation methodologies for comparing GO ecotoxicity results under axenic and non-axenic conditions as well as a multimarker approach to accurately evaluate the risks posed by GO.

## 1. Introduction

Graphene oxide (GO), one of the most commonly used derivatives of nanomaterials of the graphene family, is produced by oxidative exfoliation of graphite [1]. The extensive use of this carbon-based nanomaterial has stimulated research on its potential environmental impacts on the aquatic ecosystem during the last ten years [2,3,4]. The toxicity of pristine GO and its functionalized derivatives on aquatic organisms has been extensively studied, with a particular focus on its impact on *Daphnia magna*, a filter-feeding zooplankton widely used in ecotoxicity studies that is a key trophic link in aquatic ecosystem food chains [2,4,5,6,7]. Despite these efforts, numerous questions remain unanswered; additional aspects require further exploration. Recent comprehensive scientific studies that examined the effects of GO on *D. magna* have highlighted the critical importance of investigating well-characterized GO suspensions across molecular, physiological, and behavioral dimensions in relation to this organism [2,8,9,10]. It has been shown that the measured toxicity of GO in aquatic toxicity studies may be affected by multiple factors: differences in the physicochemical properties of the tested GO affect its behavior across different aquatic ecotoxicity test systems. Furthermore, variations in exposure conditions can lead to different levels of toxicity for the same GO product when comparing the results of standard single-species ecotoxicity tests with those from trophic transfer studies or microcosm experiments [11,12,13,14].

Interactions between GO and various organisms have been attributed to physical or chemical effects, resulting in cell entrapment, membrane damage, or oxidative stress. However, the primary mechanisms driving toxicity and specific material–organism interactions remain poorly understood, necessitating further research [15]. Studies have shown that the carbon/oxygen ratio in thermally reduced graphene oxide (trGO) directly influences its toxicity to *D. magna* [16], with toxicity increasing as the material becomes more reduced [15], while for multi-walled carbon nanotubes—another representative of carbon-based nanomaterials—the capacity to modulate biological activity through oxygen functionalization has been demonstrated by modifying their surface chemical properties using strong acid treatments or thermal annealing at high temperatures [17,18].

GO is highly sensitive to external energy sources, including heat, light, and X-rays. This sensitivity stems from the fact that the oxygen-containing functional groups, e.g., epoxide, carbonyl, carboxyl, hydroxyl that are attached to GO’s carbon monolayer are prone to reduction when exposed to external energy, resulting in the formation of reduced graphene oxide (rGO) [19]. Thermal in situ reduction is a popular procedure for creating reduced GO (rGO). In previous studies, different temperatures have been applied for in situ reduction of GO, ranging from 50 to 200 °C and resulting in varying the abundance of oxygen-functional groups present on the GO nanosheet surface, ultimately affecting their structural, mechanical, and optical properties [20,21].

The aforementioned facts indicate not only that GO undergoes gradual reduction over time under ambient conditions but also that the applied characterization probes alter its physicochemical properties [19], not to mention the effects of direct and intentional heat application during the different heat sterilization processes that are applied as the indispensable step of sample pretreatment in advance of ecotoxicological assays requiring axenic circumstances. The use of axenic conditions becomes essential when microbial contamination could interfere with the test organism’s responses or when the test substance may be metabolized or degraded by microbes. In such cases, sterilized forms of the test substance should be employed to ensure that observed effects are solely attributable to the chemical substance itself. For heat-sensitive compounds, tyndallization—a repeated cycle of moderate heating compared to autoclaving and cooling—can serve as an effective sterilization method that preserves chemical integrity while eliminating microbial contamination.

Therefore, a critical factor that may influence the toxicity of GO is the method of heat sterilization employed during its pretreatment before ecotoxicity testing. Heat sterilization methods, such as autoclaving, Tyndall treatment, or dry heat treatment, can alter the surface chemistry and physical characteristics of GO, potentially affecting its interactions with living organisms.

Gaining insight into how variations in GO’s structure or morphology impact its interactions with living organisms is crucial not only for gaining deeper insight into its biological hazards but also for accurately assessing and characterizing the risks associated with emerging GO-based nanomaterials. Equally important is the need to establish appropriate and standardized testing methodologies. This becomes particularly critical when comparing ecotoxicity data derived from both pristine and heat-sterilized forms of the same GO product, as variations in sample preparation methods can significantly alter their biological effects and risk profiles.

With this in mind, we investigated the ecotoxicity of a well-characterized GO product in its pristine and heat-sterilized forms using a variety of test methods, including conventional lethality and immobilization assays, along with physiological endpoints such as feeding activity and heartbeat rate in *D. magna*. The changes in GO’s physicochemical properties caused by two generally applied wet heat sterilization methods (autoclaving and Tyndall treatment) were thoroughly examined. Biological indicators of the oxidative stress response, like reactive oxygen species production (ROS), glutathione peroxidase (GPx), and glutathione-S transferase (GST) specific enzyme activities, were also determined to identify the antioxidative mechanisms triggered by exposure to GO. In summary, the goal of this research was to emphasize the importance of employing suitable testing and evaluation methodologies when comparing results obtained under axenic and non-axenic conditions in the context of GO ecotoxicity assessment.

## 2. Materials and Methods

### 2.1. Synthesis and Characterization of Pristine GO Nanoparticles

The synthesis method of the tested GO nanoparticles has been previously described [22]. GO nanoparticles were synthesized using the Hummers’ method from natural graphite “(Graphite Týn, Týn nad Vltavou, Czech Republic; average particle size 63 µm) with a yield of 33% [23]. To reach neutral pH, successive centrifugation (Jouan BR4i Multifunction Centrifuge, Thermo Scientific, Waltham, MA, USA; 9000 min^−1^) and thorough washing steps were applied with 1 M HCl solution and distilled water. The GO concentration of the stock suspension was ~1 *w*/*w*%. The C/O ratio and the surface area of the freeze-dried GO monolith were determined to be 2.6 and 20 m^2^/g, respectively, and were found to be thermally stable below 200 °C according to the TG/MS results.” [24]. The integrity of the aromatic graphene layers was significantly damaged by Hummers’ exfoliation. “The intensity ratio of the characteristic G (graphitic) and D (defect) peaks, *I_G_*/*I_D_* in the Raman dropped from 6.0 ± 0.5 in the pristine graphite to 1.18 ± 0.01.” [24].

### 2.2. Applied Wet Heat Sterilization Methods to the Pristine GO Suspension

Before environmental toxicological testing, distilled water was used to dilute the pristine GO suspension to 100 mg/L. One-third of the pristine 100 mg/L of GO suspension was left untreated (untreated GO), another third was autoclaved in 250 mL screw-cap laboratory bottles at 121 °C and 1.2 bar pressure for 10 min (autoclaved GO), and the remaining third underwent tyndallization (tyndallized GO). During the tyndallization process, the pristine GO suspension was treated in 250 mL screw-cap laboratory bottles with flowing steam at 100 °C for 30 min on three consecutive days. Both the pristine and heat-sterilized GO suspensions were kept in the dark at room temperature until they were used for ecotoxicity testing. Prior to any further analyses, the graphene oxide suspensions were subjected to sonication in an ice bath using a BRANSON 450 instrument (400 W, 30% amplitude) for a duration of 30 min.

### 2.3. Physicochemical Characterization of Heat-Sterilized GO Suspensions and Assembled Ecotoxicity Test Systems

#### 2.3.1. Measurements of Electric Conductivity and pH

The pH of the assembled test systems (WTW pH 330 instrument, Weilheim, Germany, Sentix 81 electrode) and electric conductivity (Consort C535 instrument) were measured at 24 and 48 h exposure times in triplicate.

#### 2.3.2. UV–Vis Spectroscopy of GO Suspensions

Optical absorbance measurements were performed on untreated, autoclaved, and tyndallized stock suspensions using a Specord 200 (Analytik Jena, Jena, Germany) UV–Vis Spectrophotometer. Data were collected in the entire UV–Vis range (190–1100 nm).

#### 2.3.3. Dynamic Light Scattering Method Characterization and Zeta Potential Determination

Untreated and heat-sterilized GO stock suspensions in distilled water, as well as samples from assembled ecotoxicity test systems (in Daphnia growth medium) at the beginning of the experiments, were analyzed in terms of intensity-weighted mean hydrodynamic particle size, polydispersity index (PDI), and Zeta potential at 25 °C using a dynamic light scattering device (Zetasizer ProBlue, Malvern Panalytical Ltd., Worcestershire, UK) equipped with a Malvern ‘Dip’ cell (ZEN1002) to measure all properties on the same sample in triplicate.

#### 2.3.4. Thermogravimetry/Mass Spectrometry

Thermogravimetry coupled with mass spectrometry (TG/MS) analyses were carried out using a modified Perkin-Elmer TGS-2 thermobalance connected to a HiQuad quadrupole mass spectrometer(Pfeiffer Vacuum, Asslar, Germany). Samples of approximately 1.5–2 mg were heated in argon (flow rate 140 mL/min) from room temperature to 250 °C at 2 °C/min, followed by a ramp of 20 °C/min up to 900 °C. A fraction of the evolved gases was directed into the mass spectrometer through a glass-lined metal capillary maintained at 300 °C. The mass spectrometer’s ion source was operated at an electron energy of 70 eV. Ion signal intensities were normalized with respect to the sample mass and the ^38^Ar isotope of the carrier gas.

### 2.4. Applied Ecotoxicity Test Methods

#### 2.4.1. *Daphnia magna* Cultures

“The in-house *D. magna* colony used for the ecotoxicity tests was cultured in 2 L beakers at 21.5 ± 1 °C in a thermostatic chamber with a 16:8 h light:dark cycle (illumination: Juwel Aquarium, Day-Lite(Rotenburg/Wümme, Niedersachsen, Germany), 15 W, 438 mm lamp, 560 Lumen, 6500 K). The *D. magna* colony was fed three times a week ad libitum with 2.0 mg DW/L of *Chlorella kessleri* (from the Mosonmagyaróvár Algal Culture Collection (MACC) of the Széchenyi István University, Department of Plant Sciences, Mosonmagyarovar, Hungary). For the maintenance of *D. magna,* boiled and cooled tap water was used.” [24]. The sensitivity of the *D. magna* colony was checked using potassium dichromate (K_2_Cr_2_O_7_) as a reference toxicant in 6-month intervals. The range of sensitivity of the *D. magna* colony to K_2_Cr_2_O_7_ was in line with the sensitivity limits (EC_50_, 24 h = 0.6–2.1 mg/L) set by the OECD (Organization for Economic Co-operation and Development) 202 guideline [25].

#### 2.4.2. *Daphnia magna* Lethality and Immobilization Assay

The acute lethality and immobilization tests of *D. magna* were performed according to the OECD 202 test guideline [25]. The nominal concentrations tested for the untreated, autoclaved, and tyndallized GO suspensions were 3.125, 6.25, 12.5, 25, and 50 mg/L. Distilled water was applied as the negative control.

#### 2.4.3. *Daphnia magna* Heart Rate Test

To determine the time- and concentration-dependent effects of the untreated, autoclaved, and tyndallized GO suspensions, ten non-pregnant, 10-day-old *D. magna* individuals (not from the first brood) were exposed to nominal concentrations of 3.125, 6.25, 12.5, 25, and 50 mg/L GO in three replicates. The volume of each assembled test system containing ten daphnids was 50 mL, and feeding did not occur during the test. Distilled water was used as a control. The dissolved O_2_ concentration was monitored with a WTW ‘Portable Meter 340i instrument at the beginning and end of the test, recommended by the OECD 202 guideline to be at least 3 mg/L. The incubation was carried out under the conditions described in Section 2.4.1. After 24 and 48 h of exposure, each test individual was carefully placed on a single-cavity microscope slide in a single droplet (~30 µL) of test suspension with the help of a 10 mL volume automatic pipette with a cut pipette tip to avoid damage or disturbance of the daphnids due to the original small cross-sectional area of the tip. The heart rate of the daphnids was recorded for 10 s but was important within a maximum of 30 s after placing them on the microscope slide under a NIKON SMZ800 stereomicroscope (32× magnification) [24].

#### 2.4.4. *Daphnia magna* Feeding Activity Inhibition Test

Feeding activity was measured according to the method of Kamaya et al. [26] with major modifications. The assembly of the test systems and the GO concentrations tested are identical to those described in the case of the heart rate test (Section 2.3.3).

“After 24 and 48 h of exposure to the tested GO samples, the groups of ten daphnids were transferred to 10 mL of a 3 µL/mL concentration fluorescent microsphere suspension (Life Technologies (Carlsbad, CA, USA); FluoSpheres™ Carboxylate-Modified Microspheres, 0.2 µm, yellow-green fluorescent (505/515), 2% solids) diluted with the original *D. magna* growth medium. After a 20 min period in the fluorescent microsphere suspension, the daphnids were taken out with a special fabric spoon and washed thoroughly with distilled water to remove the microspheres adhered to their carapace and appendages to avoid bias of the results and to detect only the amount of microbeads that are released from the digestive tract of the daphnids due to sonication. The washed individuals were transferred to micro test tubes containing 1 mL of distilled water, then homogenized for 5 s with Sonoplus HD 4100 homogenizer (BANDELIN electronic GmbH & Co. KG, Berlin, Germany) with the following settings: frequency: 19,800 Hz, amplitude: 30%. The homogenized Daphnia-microsphere suspensions were pipetted into four parallel wells of a white 96-well flat-bottom microtiter plate and the fluorescence intensity of the wells was measured by the FLUOstar Optima microplate reader (BMG LABTECH GmbH, Ortenberg, Germany) using the excitation wavelength of 485 nm and the emission wavelength of 520 nm” [24]. Feeding activity inhibition [%] was calculated relative to the fluorescence intensity of the control group.

### 2.5. Biomarkers of Oxidative Stress

Before the biochemical assays, ten daphnids of 24 h exposure samples were homogenized in 1 mL of phosphate-buffered saline (PBS; pH 7.4) using a Teflon tissue grinder. The homogenates were transferred to 1.5 mL microcentrifuge tubes and centrifuged at 14,000× *g* for 15 min at 4 °C. The total protein content and activity of the antioxidant enzymes glutathione peroxidase (GPx) and glutathione-S-transferase (GST) were measured in the supernatants. PBS (pH 7.4) was used as a blank to analyze the production of antioxidant enzymes with a Thermo Scientific Multiskan GO UV/Vis microplate reader with Thermo Scientific SkanIt Software 6.0.1 software. The supernatants were stored on ice during the measurements.

The total protein content of the samples was measured using the Pierce™ BCA Protein Assay Kit from Thermo Scientific based on the Bradford method [27], with bovine serum albumin as the standard.

GPx activity was determined using the method of Paglia and Valentine [28]. A total of 18 µL supernatant was added in triplicates to 160 µL reagent buffer (pH 7.0; 5 M K_2_HPO_4_, 5 M KH_2_PO_4_, 3.5 mM reduced glutathione, 1 mM sodium azide, 2 U glutathione reductase, and 0.12 mM NADPH) and 70 µL of 20 mM H_2_O_2_ with an electronic multichannel pipette with 12 channels to minimize time delay when measuring the reaction kinetics. The absorbance was measured at 340 nm for 60 s. One unit of GPx activity corresponded to the production of 1 µM glutathione disulfide (GSSG) per minute (ε^mM^ = 6.22).

GST activity was analyzed following Habig et al. [29]. Before the test, a 3 mM 1-chloro-2,4-dinitrobenzene (CDNB) solution was prepared by dissolving it in 15 mL of ethanol and diluting it to 250 mL with 0.1 M potassium phosphate buffer (pH 6.5) to achieve the final concentration. Reduced glutathione (RG) was also dissolved in PBS (pH 6.5) at a concentration of 0.01 M. Then, 15 µL supernatant was added in triplicates to a 96-well microtiter plate, then mixed with 195 µL RG PBS solution, and 100 µL CDNB solution was added with an electronic multichannel pipette with 12 channels to minimize the time delay when measuring reaction kinetics to initiate the reaction. The absorbance was measured at 340 nm for 1 min. One unit of GST activity was defined as the amount of enzyme that catalyzes glutathione conjugation with 1 nM CDNB per minute (ε^mM^ = 9.6).

ROS production was evaluated using the method described by Ulm et al. [30], with modifications. The assay used 2′,7′-dichlorodihydrofluorescein diacetate (DCFH-DA), a non-fluorescent probe that becomes fluorescent upon oxidation by intracellular ROS. A total of 50 µL of Daphnia homogenate supernatant was measured in a white microtiter plate in triplicate, followed by the addition of 200 µL of PBS 7.4 buffer containing DCFH-FDA. The final concentration of DCFH-FDA was 10 µM in the wells. The fluorescence intensity (FI) of the wells was measured with a FLUOstar Optima microplate reader (BMG LABTECH GmbH, Ortenberg, Germany) using an excitation wavelength of 485 nm and an emission wavelength of 520 nm after 2 h of incubation at room temperature in the dark. Data are expressed as a percentage of fluorescence compared to the relevant negative controls.

### 2.6. Data Evaluation and Statistical Analysis

Inhibition percentages (H%) were determined relative to the control for each ecotoxicological endpoint. Statistical analyses were conducted using one-way ANOVA and factorial ANOVA in STATISTICA 13.1^®^ (TIBCO Software, Inc., Palo Alto, CA, USA) to identify significant effects (*p* < 0.05). The homogeneity of variances was assessed with Cochran’s C test. Significant differences between treatments or dilutions were evaluated using the Newman–Keuls test (*p* < 0.05). In figures, alphabetical letters denote statistically significant differences, with ‘a’ representing the lowest mean value; treatments sharing the same letter are not significantly different. Effective concentration values (EC_20_ and EC_50_) were calculated in Origin^®^ 2018 (OriginLab, Northampton, MA, USA) by fitting the data to a logistic function: y = A_2_ + (A_1_ − A_2_)/(1 + (x/x_0_)^p^).

## 3. Results and Discussion

### 3.1. Physicochemical Characterization of GO Suspensions and the Assembled Ecotoxicity Test Systems

The pH of the assembled test systems ranged from 7.74 to 8.23, whereas their electrical conductivity varied between 784 and 822 µS/cm (Table S1).

The applied sterilization methods can be considered as hydrothermal treatments of the GO under different conditions. Autoclave treatment is short and takes place at higher temperature and pressure than tyndallization, while tyndallization is a much longer and repeated procedure. First, we studied the impact of these sterilization procedures on GO itself. According to Huang et al. [31], the hydrothermal treatment of GO results in the slow reduction of the nanoparticle. When the effect of the sterilized GO is studied, we have to consider two effects: the change of the nanoparticle and the change of the medium, as the non-volatile products of the GO alteration remain in the aqueous phase.

The effect of the sterilization treatments autoclaving and tyndallization on the GO in an aqueous medium was revealed by UV–Vis spectroscopy. Figure S1 shows the spectra before and after the treatments. We followed the sharp absorption peak at 228 nm and the broad shoulder at ~290–305 nm, as seen in Figure S1. The absorption peak at 228 nm belongs to the π⟶π* transition of the C=C bonds, while the broad shoulder in the range 280–305 nm is assigned to the n⟶π* transitions of the carbonyl groups [23]. Absorbance values were read at 228, 294, and 600 nm. The latter can be attributed to the scattering of the particles in the suspension. The absorbances were normalized to the π⟶π* peak (228 nm). During the treatments, the n⟶π*/π⟶π* absorbance ratios increased, indicating that the sterilization treatments led to the formation of additional C=O groups, e.g., from carboxylic groups. Under the treatment conditions, the particles may aggregate, increasing the scattering in the visible region. The sterilization treatments also resulted in the darkening of the suspensions, which is a sign of reduction.

**Figure 1:**
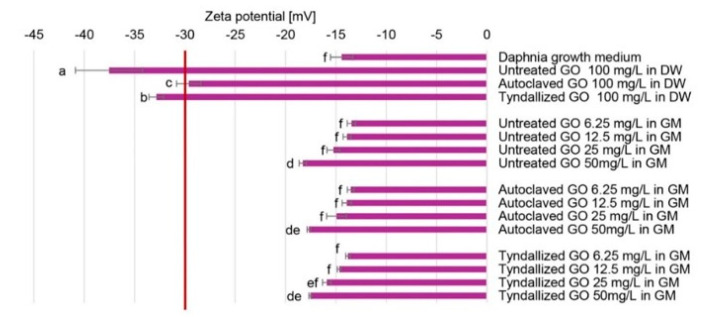
Zeta potential values measured in the GO stock suspensions and the assembled test systems reported from *n* = 3 determinations per sample. (DW: distilled water; GM: growth medium). Significant differences (*p* < 0.05) are indicated by lowercase letters, where ‘a’ represents the lowest mean value. Values marked with the same letter did not show significant differences.

Zeta potential values beyond the range of −30 mV to +30 mV are generally considered sufficient to provide repulsive forces that enhance the physical stability of colloidal systems. Conversely, low Zeta potential values may lead to particle aggregation and flocculation due to van der Waals attractive interactions [32]. According to the Zeta potential values shown in Figure 1, all GO stock suspensions in distilled water proved stable (−37.5 +/− −3.4 to −29.6 +/− −1.2 mV). However, there were slight differences in their Zeta potential values. The untreated GO stock suspension seemed to have the strongest colloidal stability among the three stock suspensions. Both sterilization procedures reduced the Zeta potential, although there was a subtle difference in their effect. This finding aligns well with the UV–Vis results, as the reduction of the particles decreases their hydrophilicity, and consequently, their colloidal stability. The Zeta potential values measured for the GO suspensions diluted with the growth medium (GM) were generally lower, which can be attributed to the compression of the electric double layer resulting from the increased ionic strength. These values (−18.3 +/− −0.4 to −13.5 +/− −0.4 mV) suggest the limited colloidal stability of these suspensions; therefore, aggregation of GO particles can be anticipated in the Daphnia growth medium. The concentration-dependent tendency of the Zeta-potential values (Figure 1) in the case of the untreated, autoclaved, and tyndallized GO suspensions is highly similar, possibly because the effect of ionic strength is much stronger than the sterilization methods applied.

Although intensity-weighted mean hydrodynamic size (Z-average) and polydispersity (PDI) were determined for the GO stock suspensions and their growth medium-diluted versions at different concentrations, it is important to note that the method is useful for spherical particles, but its application is more challenging for heterodisperse and anisotropic systems like GO suspensions. Therefore, no further conclusions were drawn from the results obtained. The hydrodynamic diameter distribution results (Figure S4) of the Daphnia growth medium, the GO stuck suspensions in distilled water (DW), and samples from the assembled test systems with GM suggest a large fraction of particles in the range of 1–2.5 µm, in many cases with a multi-peaked (highly heterodisperse) size distribution, so a clear trend could not be observed. Smaller sizes were only seen in the case of GO stock suspensions (mostly below 1 µm).

**Figure 2:**
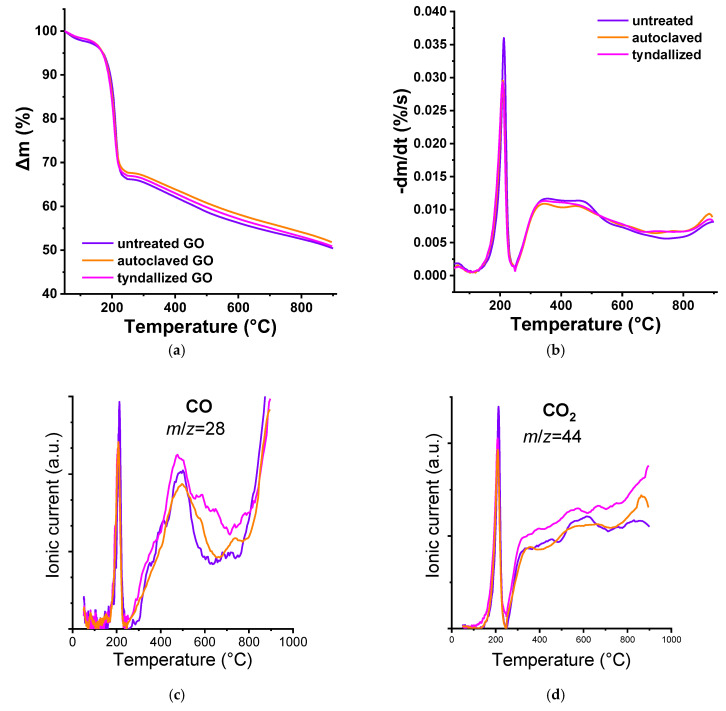

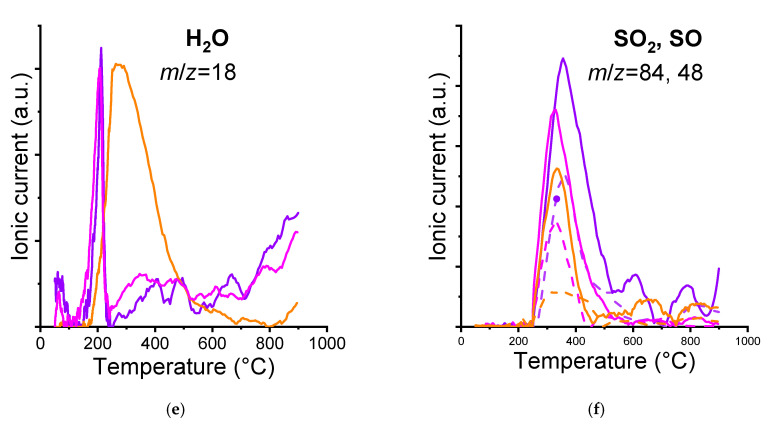
TG/MS analysis results of the pristine and heat-sterilized GO suspensions: Δm (**a**), -dm/dt (**b**), CO fragment (**c**), CO_2_ fragment (**d**), H_2_O fragment (**e**). In the case of the SO_2_/SO diagram (**f**), the solid line represents SO_2_ and its decomposition product, while the dashed line represents SO.

TG/MS analysis was also applied to follow the effect of the heat sterilization treatments. The TG and DTG results of the freeze-dried samples (Figure 2a,b) revealed that there was only a slight difference between the curves. TG/MS was used to follow the low *m*/*z* decay fragments listed in Table 1 to shed light on the source of the differences. Water, CO, and CO_2_ were the main components. The source of SO_2_ and its derivative fragment SO between 300–400 °C stems from the sulfuric acid retained after a modified Hummers exfoliation (Hummers et al., 1958 [23]). The temperature range in which CO (*m*/*z* = 22) and CO_2_ (*m*/*z* = 44) fragments appear can be correlated with the decomposition of functional groups decorating the carbon nanoparticles [33]. In our case, the temperature ramp of 20 °C/min was higher than the 5–10 °C/min usually employed in the TPD experiments; the temperature ranges may appear at slightly higher values than reported in the reference works.

From the pristine GO sample, water is eliminated up to 200 °C, as is also CO_2_ from carboxyl groups. Isolated carboxyls are released directly upon heating; those having adjacent carboxylic groups can undergo dehydration.

The decomposition of anhydride groups occurs between 350 and 600 °C, releasing equal amounts of CO and CO_2_ molecules. CO_2_ above 600 °C derives from the lactone groups, which may also result from the condensation of the adjacent carboxyl and phenol groups. Anhydrides or lactones are not necessarily present in GO but may be formed during heat treatment. Although no definitive CO band can be attributed to phenol groups (500–750 °C), the CO release above 800 °C implies the presence of carbonyls/quinones (Haydar et al., 2000 [34]). Pyrone-type groups that release CO above 950 °C could be present or develop during heat treatment, but that range lies outside of our TG/MS measurements. As a result of the Tyndall treatment, the concentration of phenolic functional groups in the sample increased compared to the pristine GO, as indicated by the differences observed in the 500–800 °C range. In contrast, autoclaving did not induce such changes (Figure 2c).

Although both the TG/DTG and TG/MS results of the tyndallized samples were very similar to the untreated pristine GO, the autoclave treatment resulted in some alteration, although the run of the CO and CO_2_ curves revealed not much change. In addition, SO_2_ and SO signals were still present, but in lower concentration.

The pristine GO suspension contained residual sulfuric acid originating from the synthesis, as trace amounts are generally retained. The concentration of these residues decreased following the Tyndall treatment and was further reduced after the autoclaving. Therefore, the component that is absent from our processed materials but present in the exposure medium likely contributes to the differential toxicity observed in *Daphnia magna* (Figure 2f).

More striking is the shift and widening of the originally sharp water peak. The water signal in the 150–450 °C range may derive from the dehydration of adjacent carboxylic groups [35]. The water released at a more elevated temperature would correspond to dehydration of phenolic species, especially from ortho hydroquinone [34] (Figure 2 and Figure S3). The pronounced H_2_O peak observed for the autoclaved sample indicates that the autoclaving the pristine GO suspension results in a high concentration of adjacent carbon atoms bearing acidic protons (i.e., –carboxyl functional groups). Such groups do not form during the Tyndall treatment, nor were they present in the original sample (Figure 2e).

### 3.2. Results of the Daphnia magna Lethality and Immobilization Tests

Table 2 summarizes the results of the *D. magna* lethality and immobilization tests. Based on inhibition percentage data, although an increasing trend in the toxicity of untreated, autoclaved, and tyndallized GO samples could be observed, the calculated inhibitions relative to the control group were not statistically significant. Lv et al. [36] tested the effect of a GO product in the 1–50 mg/L concentration range with physicochemical characteristics different from the GO used in our study and reported similar results of lethality and immobilization, as in our case, with a time and concentration-dependent toxicity. The GO-exerted toxicity can be considered relatively moderate based on the lethality and immobilization results of our study and the results of Lv et al. [36]; on the contrary, other types of GOs synthesized by the Hofmann, Hummers, and Tour protocols that resulted in three different oxidation levels exerted more severe toxicity on D. *magna* with ~25–50% lethality after 48 h exposure in 25 mg/L concentration [10]. These results highlight the potential variability in GO toxicity, ranging from minimal toxic effects to 50% inhibition, which can be attributed to differences in product characteristics such as oxidation level, C/O ratio, and specific surface area.

### 3.3. Results of the D. magna Heart Rate Test

**Figure 3:**
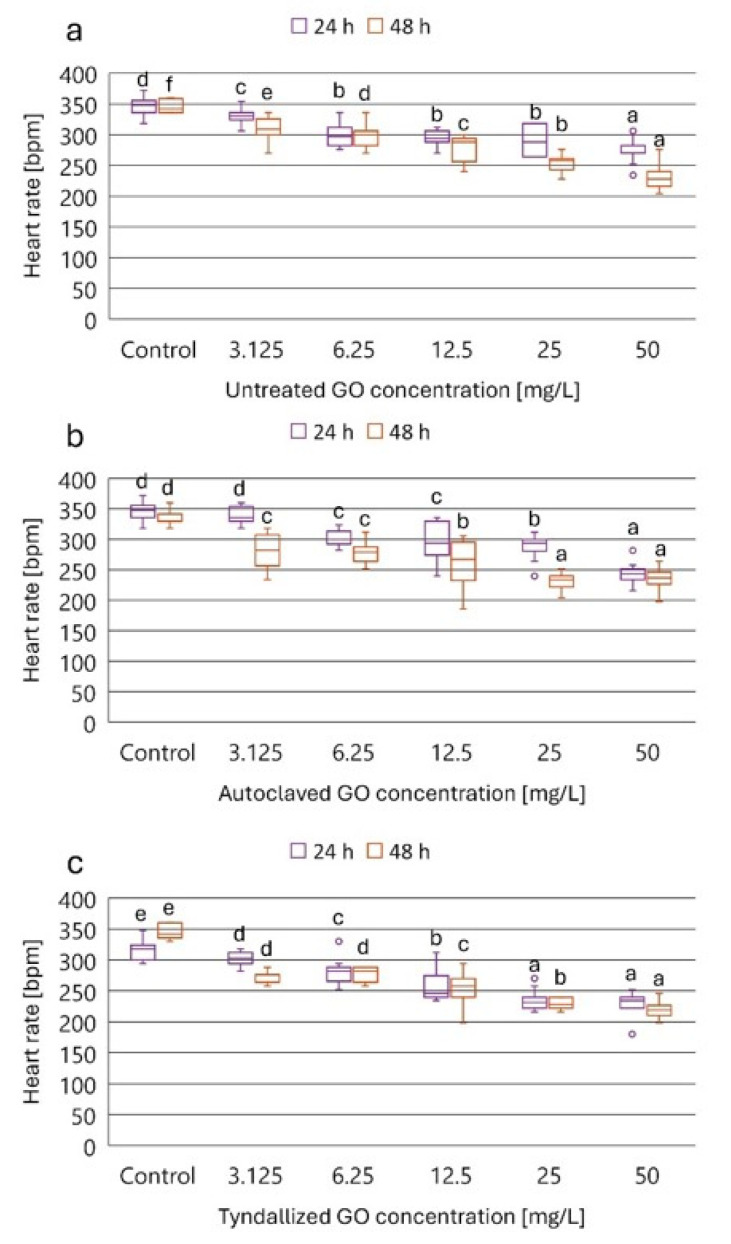
The effect of untreated (**a**), autoclaved (**b**), and tyndallized (**c**) GO on the heart rate of *D. magna* in beat per minute (bpm) units after 24 and 48 h of exposure. Significant differences (*p* < 0.05) between concentrations distinctly for each exposure time are indicated by lowercase letters, where “a” represents the lowest mean value. Treatments marked with the same letter showed no significant differences.

We aimed to determine how the GO suspensions underwent different heat sterilization procedures that affected the normal physiological processes of *D. magna*, such as heart rate, in a concentration- and time-dependent manner. The heart rate results obtained for the three GO suspensions (untreated, autoclaved, and tyndallized) are presented in Figure 3, while the corresponding inhibition percentages compared to the control group are summarized in Table 3.

Based on the inhibition percentage values summarized in Table 3, it can be concluded that the three GO samples exhibited concentration-dependent and exposure time-dependent inhibitory effects on the heart rate of *D. magna*. The lowest tested GO concentration (3.125 mg/L) resulted in 8, 6, and 12% inhibition of the heart rate by the untreated, autoclaved, and tyndallized GO, respectively, after 24 h exposure, while after 48 h exposure, the inhibitory effect was more pronounced: 14, 22, and 24%, respectively. The highest tested GO concentration (50 mg/L) resulted in 23, 32, and 34% inhibition of the heart rate by the untreated, autoclaved, and tyndallized GO, respectively, after 24 h exposure, while after 48 h exposure, the inhibitory effect of the untreated GO was considerably higher compared to the 24 h effect. In the case of the autoclaved and tyndallized GOs, inhibition at 48 h exposure was only slightly higher compared to the inhibition experienced after 24 h exposure. 14, 22, and 24%. When comparing the 48 h exposure results, it can be concluded that only a small difference could be found between the inhibitory effect of the two heat-sterilized GO samples. However, both of them exerted toxicity higher than that of the untreated GO suspension. In general, exposure to autoclaved and tyndallized GO resulted in greater inhibition of heart rate than untreated GO in *D. magna* with the exception of the highest concentration tested (50 mg/L).

While Daphnia heart rate has been used as a sensitive, sublethal physiological endpoint in several ecotoxicity studies [37,38,39,40,41], examples remain rare when it comes to characterizing nanomaterial toxicity [38,42,43,44], especially carbon-based nanomaterials (CNMs) [44] such as GO [9]. Lovern et al. [45] reported CNM-specific alterations of normal heart rate in *D. magna* in 260 µg/L concentration within a 60 min exposure period, registering the heart rate every 10 min: nano-C_60_ caused a significant increase in the average heart rate by 43.6 beats per minute, while C_60_HxC_70_Hx caused an average decrease of −4.37 beats per minute that was not statistically significant. To the best of our knowledge, modulation of Daphnia heart rate by GO has not been studied, except for our previous work on GO toxicity in *D. magna* [9]. Therefore, a direct comparison is not possible. However, it can be noted that observed changes in heart rate tend to involve slowing rather than acceleration, and complete recovery occurred in a clean test medium within 4 h, even after 48 h of exposure [9].

Free oxygen radicals, generated as a result of oxidative stress, have been observed to affect normal physiological functions, including cardiac activity, leading to a decrease in the heart rate of *D. magna* [46]. However, this effect has also been shown to be alleviated by using antioxidant agents, which neutralize ROS and reduce oxidative stress [47]. We found that exposure to GO led to a significant decrease in the normal average heart rate of *Daphnia magna*. We hypothesize that this decrease is directly related to ROS production induced by exposure to GO. Our experimental results further support this hypothesis, showing elevated ROS levels in GO-exposed daphnids compared to the control group.

### 3.4. Results of the D. magna Feeding Activity Inhibition Test

**Figure 4:**
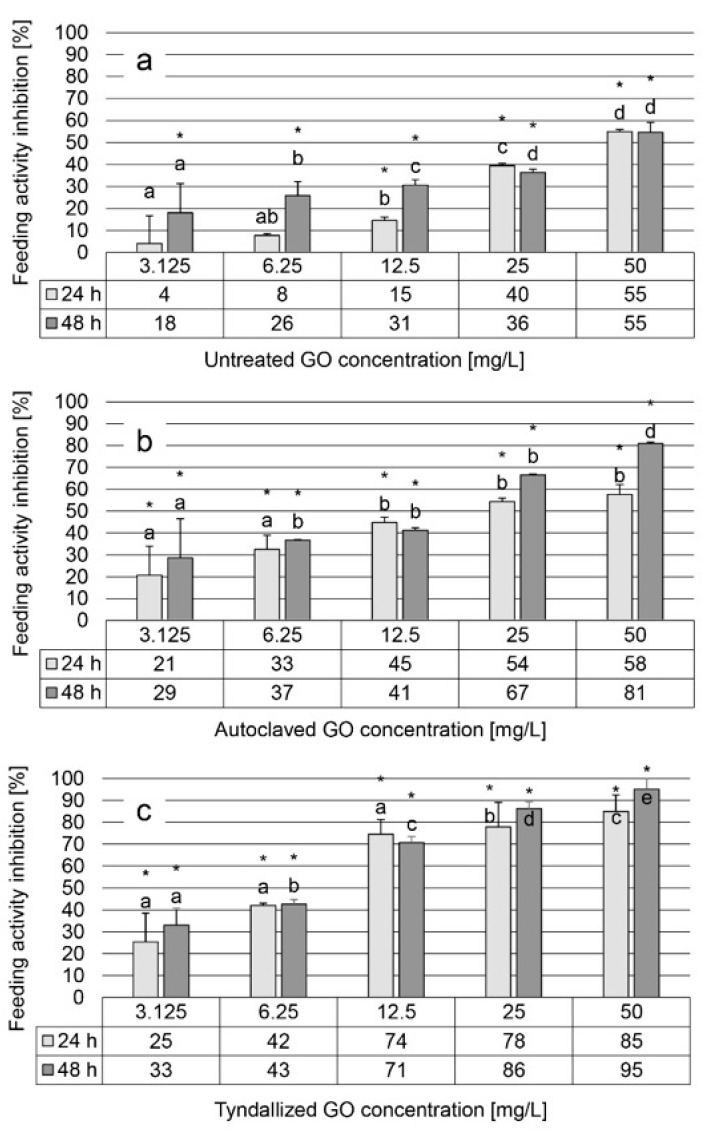
The effect of untreated, autoclaved, and tyndallized GO on the feeding activity of *D. magna* given in inhibition percentage [%] units after 24 and 48 h of exposure. Feeding activity inhibition was calculated relative to the fluorescence intensity of the control group. Significant differences (*p* < 0.05) between concentrations distinctly for each exposure time are indicated by lowercase letters, where “a” represents the lowest mean value. Treatments marked with the same letter showed no significant differences. Significant inhibition compared to control is marked by an asterisk (*).

The results of the feeding activity inhibition test showed a similar trend of inhibition across the three graphene oxide (GO) suspensions tested, comparable to the observations in the heart rate test, although with greater variability and to a greater extent. Untreated GO suspension induced 4–55% inhibition after 24 h of exposure and 18–55% inhibition after 48 h, within a concentration range of 3.125–50 mg/L, displaying a concentration-dependent effect (Figure 4a). For autoclaved GO suspension, inhibition levels ranged from 21–58% and 29–81% after 24 and 48 h, respectively (Figure 4b). On the contrary, the tyndallized GO suspension exhibited higher inhibition levels, ranging from 25–85% after 24 h to 33–95% after 48 h (Figure 4c).

**Figure 5:**
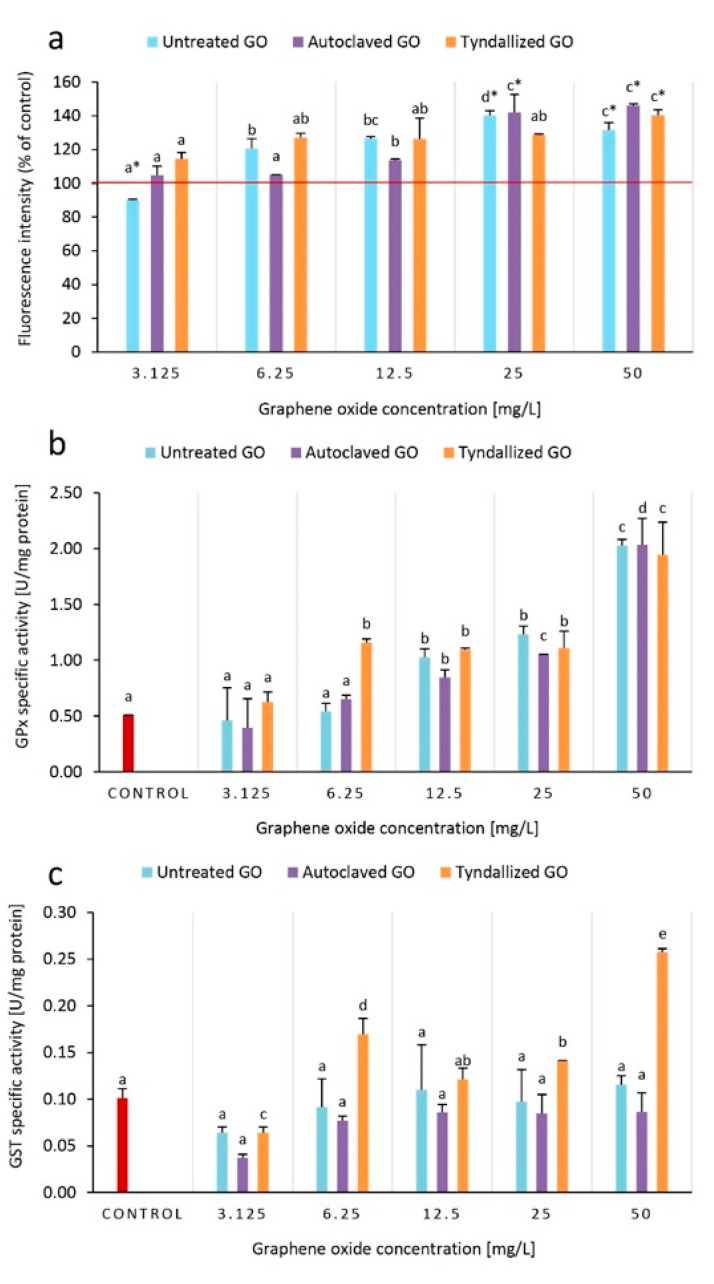
Effect of GO on ROS levels based on DCFH-DA fluorescence intensity (**a**), GPx (**b**), and GST (**c**) antioxidant enzyme activities after 24 h of exposure to GO. ROS levels are compared to the control (100%), and higher intensity than 100% corresponds to higher ROS levels. Significant differences (*p* < 0.05) between concentrations distinctly for each type of GO are indicated by lowercase letters, where “a” represents the lowest mean value. Treatments marked with the same letter did not show significant differences.

The uptake and depuration constants of GO were quantified by Lv. et al. [36] in *D. magna*, demonstrating rapid uptake and slow depuration rates of GO. Although their results showed that almost complete GO depuration can be achieved after 24 h by algae feeding, other studies reported limited GO depuration without or even with algae feeding for other types of CNMs [48,49]. Considering the physicochemical properties of these CNMs, it is likely that both facilitated uptake and easier depuration of GO may be attributed to its high hydrophilicity, hence enhanced dispersibility in the aqueous test system, as hydrophobic nanomaterials tend to have stronger interaction with the inner surface of the digestive tract and are more easy to purge due to stronger aggregation [50,51]. In vivo fluorescent visualization of the location of GO-induced oxidative stress was found to be mainly in the digestive tract of *D. magna*, both confirmed by Lv et al. [36] and Fekete-Kertész et al. [9], suggesting that direct contact of accumulated GO with the inner lining of the digestive tract negatively affects the test organism. Our results of the GO concentration-dependent elevated ROS production (Figure 5) also support the hypothesis that there is a direct relationship between oxidative stress and the decline of normal feeding activity.

Another possible contributing factor to GO-induced feeding inhibition may be the impairment of swimming velocity based on the physical restriction of movements due to adhered GO nanoparticles to antennae, ultimately affecting predator–prey interactions [8]. Alterations of the thoracic appendages’ movement amplitude due to physical or chemical adverse effects of GO are also likely to inhibit filtration by interfering with the filter-feeding mechanism [52].

### 3.5. Results of the Oxidative Stress Biomarkers

To unravel the underlying mechanism of GO toxicity on *D. magna*, ROS production, and two scarcely studied oxidative biomarkers in the case of GO ecotoxicity, the activities of the GPx and GST enzymes were determined after 24 h of exposure to GO.

Compared to the control group, fluorescence intensity (FI) increased in the groups exposed to untreated GO concentrations of 6.25–50 mg/L, while in the case of autoclaved GO, an increase in FI was observed only in the concentrations of 12.5–50 mg/L. The tyndallized GO sample increased ROS production in all concentrations tested (3.125–50 mg/L) (Figure 5a). GPx enzyme activities were significantly higher in tyndallized GO treated groups (6.25–50 mg/L), while in the case of the untreated and autoclaved GO samples GPx activity was increased only in the 12–50 mg/L concentration range. In general, GPx activity showed a concentration-dependent increase in relation to GO concentrations (Figure 5b). The specific GST enzyme activity did not show a clear trend. However, it can be noted that the strongest increase in activity was experienced in the Tyndallized GO-treated groups.

GO-induced oxidative stress in *Daphnia* sp. was previously investigated. However, these studies provided inconclusive results due to the varying characteristics of GO and exposure conditions [8,10]. The regulation of antioxidative processes at the cellular level is a complex mechanism with a highly sensitive equilibrium of ROS production and elimination in the system of a living organism [53]. Oxidative stress biomarkers are commonly used in ecotoxicology, but identifying the specific causes of observed biochemical changes can be challenging due to the multitude of factors that influence an organism’s oxidative state [54]. Excess oxidative stress can exceed the capacity of detoxifying or antioxidant systems, leading to substantial oxidative damage and a breakdown of compensatory mechanisms [36]. GO-induced alterations in ROS scavenger mechanisms [53] may result in macromolecular damage leading to cell, tissue, and ultimately, organ dysfunction in daphnids [55], as was experienced in the case of our experiments based on cardiac dysfunction and inhibited feeding activity. It has been shown that variability in the feeding rate itself can significantly influence oxidative stress biomarkers in *D. magna*, and combining the effects of feeding rate and exposure to xenobiotics on oxidative biomarkers in invertebrates remains highly understudied [54,56]. It should be noted that experienced inhibition of the feeding rate by exposure to GO can affect the interpretation of measured biomarker responses, highlighting the challenges of understanding differences in metabolic state, feeding rate, and exerted toxicity by GO itself.

While there are some examples in the literature of the effects of sterilization methods on the properties and stability of nanomaterials [57,58], there are no examples of such tests specifically for GO. The results of these studies clearly show that sterilization processes can affect the physicochemical properties (e.g., polydispersity index, zeta potential, mean particle size) of different types of nanoparticles. However, the current evidence base remains limited, both methodologically and in terms of the available datasets, which restricts the extent to which comprehensive modeling or broader generalizations can be reliably made. Therefore, further refinement of the physicochemical characterization techniques is necessary to reveal subtle nuances in all relevant properties of individual GO samples, including those detectable only with high-resolution methods such as TEM or SEM. Even minor, potentially undetectable physicochemical changes may collectively influence toxicity outcomes in a synergistic manner. Importantly, these effects are also shaped by experimental conditions and composition of the test medium used in the ecotoxicological assays, underscoring the need for GO-by-GO product-specific assessment. The relevance of our research extends beyond the field of ecotoxicology, including the need for sterilization of nanomaterials in biomedical applications [57,58].

### 3.6. Comparative Evaluation of the Ecotoxicity Endpoint Sensitivity and GO Toxicity

Relatively high GO exposure concentrations (3.125–50 mg/L) were used to determine effective concentration values (EC_20_ and EC_50_), facilitating the comparison of standard and innovative endpoint sensitivities (Table 4). A comparative statistical evaluation was performed with the results of each applied ecotoxicological endpoint: lethality, immobilization, heart rate, and feeding activity (Table S2). As anticipated, conventional standardized lethality and immobilization assays were the least sensitive among all ecotoxicity tests with *D. magna*. In contrast, the innovative sublethal ecotoxicity endpoints demonstrated greater sensitivity, which underscored the importance of refined, more sensitive sublethal endpoints in nano-ecotoxicology. The most sensitive endpoint was found to be feeding activity, followed by heart rate.

The EC_20_ and EC_50_ values allow for a direct comparison of the toxic effects of the three GO suspensions (untreated, autoclaved, and tyndallized) (Table 4). Among the two most sensitive endpoints, the feeding activity test EC_20_ values indicate that the tyndallized GO was the most toxic at both exposure times (EC_20,24h_ = 2.59 mg/L, EC_20,48h_ = 1.95 mg/L), followed by the autoclaved GO (EC_20,24h_ = 3.24 mg/L, EC_20,48h_ = 2.92 mg/L), and lastly the untreated GO (EC_20,24h_ = 18.62 mg/L, EC_20,48h_ = 5.61 mg/L). For the heart rate ecotoxicity endpoint, the toxicity ranking was consistent with that determined for the feeding activity test, with the tyndallized GO being the most toxic (EC_20,24h_ = 29.01 mg/L, EC_20,48h_ = 2.03 mg/L), followed by the autoclaved GO (EC_20,24h_ = 18.07 mg/L, EC_20,48h_ = 16.17 mg/L), and the untreated GO being the least toxic (EC_20,24h_ = 36.49 mg/L).

## 4. Conclusions

A direct relationship between the effect of heat sterilization of GO nanoparticles and their exerted toxicity has been revealed in the *Daphnia magna* ecotoxicity test system with a multimarker approach from the molecular to physiological levels, including behavioral changes. Based on the corresponding effective concentration values, heart rate and feeding activity proved to be significantly more sensitive ecotoxicity endpoints than conventional lethality and immobilization tests. Elevated levels of ROS production and an increase in the antioxidative mechanisms (GPx and GST enzyme activities) were experienced due to exposure to GO. However, a direct correlation between the antioxidant mechanisms investigated and the physiological changes observed cannot be conclusively established on the basis of the ecotoxicological findings. This highlights the complexity of linking biochemical markers with the system-level responses of an organism. Based on the results of the physicochemical characterization, only minor differences were observed between the pristine GO suspension and the two heat-sterilized samples. These differences do not show a clear correlation with toxicity. It is likely that additional exfoliation and oxidation occur during the applied heat sterilization procedures, resulting in changes that cannot be adequately characterized using the analytical methods employed. However, the toxicity characterization of systematically modified GO products could be a feasible approach to reveal structure–property–toxicity correlations; it does not provide a solution to deduce the obviously altered biological effects based on subtle physicochemical changes due to sample preparation procedures. Therefore, further refinement of the physicochemical characterization techniques is necessary to reveal nuances in all physicochemical characteristics of different GO samples that together may alter their toxicity in a synergistic way. Taking into account these factors, an approach to GO-by-GO ecotoxicity characterization is recommended, and ecotoxicity test results should prevail over toxicity predictions based on GO characteristics. Our study also highlights the critical relevance of the applied ecotoxicological testing methodology for GO, highlighting that without a clear understanding of the structure–property–toxicity relationships of materials, the application of generalized safe design guidelines can inadvertently increase ecological hazards. The relevance of our research extends beyond the field of ecotoxicology, including the need for sterilization of nanomaterials in biomedical applications.

## Figures and Tables

**Table 1:** Low *m*/*z* decay fragments of the TG/MS analysis.

*m*/*z*	Fragment
16	CH_4_
18	H_2_O
28	CO
44	CO_2_
48	SO
64	SO_2_

**Table 2:** Inhibition percentage values [%] of untreated, autoclaved, and tyndallized GO suspensions on survival (lethality %) and mobility (immobilization %) of *D. magna* relative to the control group after 24 and 48 h of exposure.

Lethality [%]						
	Untreated GO		Autoclaved GO		Tyndallized GO	
Conc. [mg/L]	24 h	48 h	24 h	48 h	24 h	48 h
3.125	0 ± 0	0 ± 0	0 ± 0	0 ± 0	0 ± 0	3 ± 6
6.25	0 ± 0	0 ± 0	0 ± 0	0 ± 0	0 ± 0	7 ±6
12.5	0 ± 0	0 ± 0	3 ± 6	3 ± 6	3 ± 6	7 ± 6
25	0 ± 0	0 ± 0	0 ± 0	7± 6	7 ±12	10 ± 10
50	0 ± 0	10 ± 0	0 ± 0	17 ± 6	7 ± 6	13 ± 15
**Immobilization [%]**						
	**Untreated GO**		**Autoclaved GO**		**Tyndallized GO**	
**Conc. [mg/L]**	**24 h**	**48 h**	**24 h**	**48 h**	**24 h**	**48 h**
3.125	0 ± 0	0 ± 0	0 ± 0	0 ± 0	3 ± 6	3 ± 6
6.25	0 ± 0	0 ± 0	0 ± 0	0 ± 0	0 ± 0	7 ±6
12.5	0 ± 0	0 ± 0	3 ± 6	3 ± 6	3 ± 6	7 ± 6
25	0 ± 0	0 ± 0	10 ± 10	7± 6	10 ±10	10 ± 10
50	0 ± 0	10 ± 0	10 ± 0	23 ± 15	7 ± 6	13 ± 15

**Table 3:** Inhibition percentage values [%] of the untreated, autoclaved, and tyndallized GO suspensions on the heart rate of *D. magna* relative to the heart rate of the control group after 24 and 48 h of exposure. Significant differences (*p* < 0.05) between concentrations distinctly for each exposure time are indicated by lowercase letters, where “a” represents the lowest mean value. Treatments marked with the same letter showed no significant differences. Significant inhibition compared to the control is marked with bold letters.

Inhibition [%]						
	Untreated GO		Autoclaved GO		Tyndallized GO	
Conc. [mg/L]	24 h	48 h	24 h	48 h	24 h	48 h
3.125	**8 ± 6 a**	**14 ± 5 a**	6 ± 7 a	**22 ± 6 a**	**12 ± 4 a**	**24 ± 2 a**
6.25	**17 ± 5 b**	**17 ± 5 b**	**16 ± 4 b**	**23 ± 6 a**	**17 ± 6 b**	**28 ± 20 a**
12.5	**19 ± 4 c**	**23 ± 5 c**	**17 ± 8 b**	**28 ± 9 b**	**25 ± 5 c**	**31 ± 6 b**
25	**19 ± 4 c**	**30 ± 5 d**	**20 ± 6 c**	**36 ± 4 c**	**36 ± 3 d**	**36 ± 3 c**
50	**23 ± 2 d**	**36 ± 6 e**	**32 ± 6 d**	**35 ± 4 c**	**34 ± 10 d**	**39 ± 3 d**

**Table 4:** Effective concentration values (EC_20_, EC_50_) of the graphene oxide suspensions tested (untreated, autoclaved, tyndallized) in the applied ecotoxicity test systems at exposure times of 24 and 48 h.

EC_20_ [mg/L]						
	24 h			48 h		
Test Endpoint	Untreated	Autoclaved	Tyndallized	Untreated	Autoclaved	Tyndallized
Lethality	>50	>50	>50	>50	>50	>50
Immobilization	>50	40.64 ± 1.72 *a*	>50	>50	44.22 ± 8.17 *a*	>50
Heart rate	36.49 ± 1.53 *e*	18.07 ± 0.94 *c*	29.01 ± 1.8 *d*	6.87 ± 0.4 *b*	16.17 ± 0.91 *c*	2.03 ± 0.14 *a*
Feeding activity	18.62 ± 0.19 *e*	3.24 ± 0.19 *c*	2.59 ± 0.21 *b*	5.61 ± 0.28 *d*	2.92 ± 0.12 *bc*	1.95 ± 0.08 *a*
**EC_50_ [mg/L]**						
	24 h			48 h		
Test Endpoint	Untreated	Autoclaved	Tyndallized	Untreated	Autoclaved	Tyndallized
Lethality	>50	>50	>50	>50	>50	>50
Immobilization	>50	>50	>50	>50	>50	>50
Heart rate	>50	>50	>50	>50	>50	>50
Feeding activity	42.58 ± 0.43 ***d***	15.46 ± 0.93 *c*	8.32 ± 0.67 *a*	42.1 ± 2.11 *d*	13.09 ± 0.52 *b*	6.92 ± 0.28 *a*

Significant differences (*p* < 0.05) between the EC_20_ and EC_50_ values determined for the GO suspensions tested (untreated, autoclaved, tyndallized) and different exposure times within each method are indicated in italic lowercase letters, with the smallest value marked as *a*. Comparative statistical lettering is interpreted row by row in the table, which means that there is no significant difference between values marked with the same letter. Shades of red illustrate the degree of toxicity: the darker the color, the greater the toxicity.

## Data Availability

The data presented in this study are available on request from the corresponding author.

## Supplementary Materials

The following supporting information can be downloaded at: https://www.mdpi.com/article/10.3390/nano15231800/s1, Figure S1: Comparison of the UV-vis spectra of 25 mg/L GO suspensions prior and after sterilization treatments; Figure S2: Z-average values measured in the GO stock suspensions and in the assembled test systems reported from n = 3 determinations per sample. (DW: distilled water; GM: growth medium); Figure S3: TG/MS analysis results of the pristine and heat-sterilized GO suspensions; Figure S4: Particle size distribution of undiluted original GO stock suspensions (a) and of the untreated, autoclaved and tyndallized GO suspensions diluted with Daphnia magna growth medium (b); Table S1: Electric conductivity and pH values measured in the assembled test systems after 24 and 48 h exposure reported from n = 3 determinations per sample; Table S2a: Factorial ANOVA results to evaluate effects of sterilization methods and GO-concentration on the heart rate and feeding activity of Daphnia magna. Bold numbers indicate significant differences at *p* < 0.05; Table S2b: Factorial ANOVA results to evaluate effects of sterilization methods and GO-concentration on the oxidative stress parameters of Daphnia magna. Bold numbers indicate significant differences at *p* < 0.05; Table S2c: Factorial ANOVA results to evaluate effects of sterilization methods and GO-concentration on the effective concentration values of the different Daphnia magna ecotoxicity endpoints. Bold numbers indicate significant differences at *p* < 0.05.
