# Supplementary material for: Pre-Experimental Wet Heat Sterilization Alters the Ecotoxicity of Pristine Graphene Oxide Toward Daphnia magna
Source: Nanomaterials (Basel). 2025 Nov 28;15(23):1800. doi: 10.3390/nano15231800 (PMC12693538; doi:10.3390/nano15231800)
Supplement: Supplementary file 1 [file nanomaterials-15-01800-s001.zip › nanomaterials-3979071-supplementary.pdf]

Supplementary

Pre-Experimental Wet Heat Sterilization Alters the Ecotoxicity of Pristine Graphene Oxide Toward *Daphnia magna*

Ildikó Fekete-Kertész [1,*], Péter Hajdinák [2], Krisztina László [3], Anna Bulátkó [3], Viktor Podhragyai [2], Benjámin Sándor Gyarmati [4], Zoltán Molnár [5] and Mónika Molnár [1]

[1] Environmental Microbiology and Biotechnology Group, Department of Applied Biotechnology and Food Science, Faculty of Chemical Technology and Biotechnology, Budapest University of Technology and Economics, Műegyetem rkp. 3., H-1111 Budapest, Hungary; molnar.monika@vbk.bme.hu

[2] Laboratory of Biochemistry and Molecular Biology, Department of Applied Biotechnology and Food Science, Faculty of Chemical Technology and Biotechnology, Budapest University of Technology and Economics, Műegyetem rkp. 3., H-1111 Budapest, Hungary; hajdinak.peter@vbk.bme.hu (P.H.); podhragyai.viktor@vbk.bme.hu (V.P.)

[3] Surface Chemistry Group, Department of Physical Chemistry and Materials Science, Faculty of Chemical Technology and Biotechnology, Budapest University of Technology and Economics, Műegyetem rkp. 3., H-1111 Budapest, Hungary; laszlo.krisztina@vbk.bme.hu (K.L.); bulatko.anna@vbk.bme.hu (A.B.)

[4] Soft Matters Group, Department of Physical Chemistry and Materials Science, Faculty of Chemical Technology and Biotechnology, Budapest University of Technology and Economics, Műegyetem rkp. 3., H-1111 Budapest, Hungary; gyarmati.benjamin@vbk.bme.hu

[5] Department of Plant Sciences, Faculty of Agricultural and Food Sciences, Széchenyi István University, Vár Sqr. 2., H-9200 Mosonmagyaróvár, Hungary; molnar.zoltan@sze.hu

* Correspondence: fekete.kertesz.ildiko@vbk.bme.hu

Table S1. Electric conductivity and pH values measured in the assembled test systems after 24 and 48 h exposure reported from n = 3 determinations per sample.

	**Untreated GO concentration [mg/L]**								
	**Control**			**3.125**			**6.25**		
	**0 h**	**24 h**	**48 h**	**0 h**	**24 h**	**48 h**	**0 h**	**24 h**	**48 h**
pH	8.02 ± 0.02	8.04 ± 0.02	8.08 ± 0.01	8.10 ± 0.01	8.14 ± 0.01	8.14 ± 0.03	8.12 ± 0.02	8.15 ± 0.01	8.13 ± 0.01
EC [μS/cm]	804 ± 1	805 ± 3	807 ± 3	798 ± 2	799 ± 1	801 ± 3	806 ± 1	809 ± 1	811 ± 3
	**12.5**			**25**			**50**		
	0 h	24 h	48 h	0 h	24 h	48 h	0 h	24 h	48 h
pH	8.05 ± 0.02	8.02 ± 0.02	8.02 ± 0.02	7.99 ± 0.01	8.02 ± 0.02	7.97 ± 0.02	7.85 ± 0.01	7.9 ± 0.02	7.88 ± 0.01
EC [μS/cm]	817 ± 3	817 ± 1	818 ± 3	820 ± 2	822 ± 3	816 ± 1	809 ± 3	811 ± 4	814 ± 4
	**Autoclaved GO concentration [mg/L]**								
	**Control**			**3.125**			**6.25**		
	**0 h**	**24 h**	**48 h**	**0 h**	**24 h**	**48 h**	**0 h**	**24 h**	**48 h**
pH	7.94 ± 0.02	7.96 ± 0.02	8 ± 0.01	8.02 ± 0.01	8.06 ± 0.01	8.06 ± 0.03	8.04 ± 0.02	8.07 ± 0.01	8.05 ± 0.01
EC [μS/cm]	796 ± 1	797 ± 3	799 ± 3	790 ± 2	791 ± 1	793 ± 3	798 ± 1	801 ± 1	803 ± 3
	**12.5**			**25**			**50**		
	0 h	24 h	48 h	0 h	24 h	48 h	0 h	24 h	48 h
pH	7.97 ± 0.02	7.94 ± 0.02	7.94 ± 0.02	7.91 ± 0.01	7.94 ± 0.02	7.89 ± 0.02	7.77 ± 0.01	7.82 ± 0.02	7.80 ± 0.01
EC [μS/cm]	809 ± 3	809 ± 1	810 ± 3	811 ± 2	814 ± 3	808 ± 1	801 ± 3	803 ± 4	806 ± 4
	**Tyndallized GO concentration [mg/L]**								
	**Control**			**3.125**			**6.25**		
	**0 h**	**24 h**	**48 h**	**0 h**	**24 h**	**48 h**	**0 h**	**24 h**	**48 h**
pH	8.23 ± 0.57	7.93 ± 0.01	7.95 ± 0	8.21 ± 0.56	8.02 ± 0	8.03 ± 0.02	8.02 ± 0.02	8.03 ± 0.01	8.01 ± 0.01
EC [μS/cm]	794 ± 4	793 ± 3	792 ± 2	787 ± 3	784 ± 5	792 ± 3	794 ± 1	797 ± 1	799 ± 3
	**12.5**			**25**			**50**		
	0 h	24 h	48 h	0 h	24 h	48 h	0 h	24 h	48 h
pH	7.93 ± 0.02	7.90 ± 0.02	7.90 ± 0.01	7.87 ± 0.01	7.91 ± 0.02	7.86 ± 0.03	7.74 ± 0.01	7.78 ± 0.03	7.76 ± 0.01
EC [μS/cm]	806 ± 2	804 ± 3	805 ± 3	806 ± 2	811 ± 3	802 ± 3	796 ± 3	799 ± 4	798 ± 5

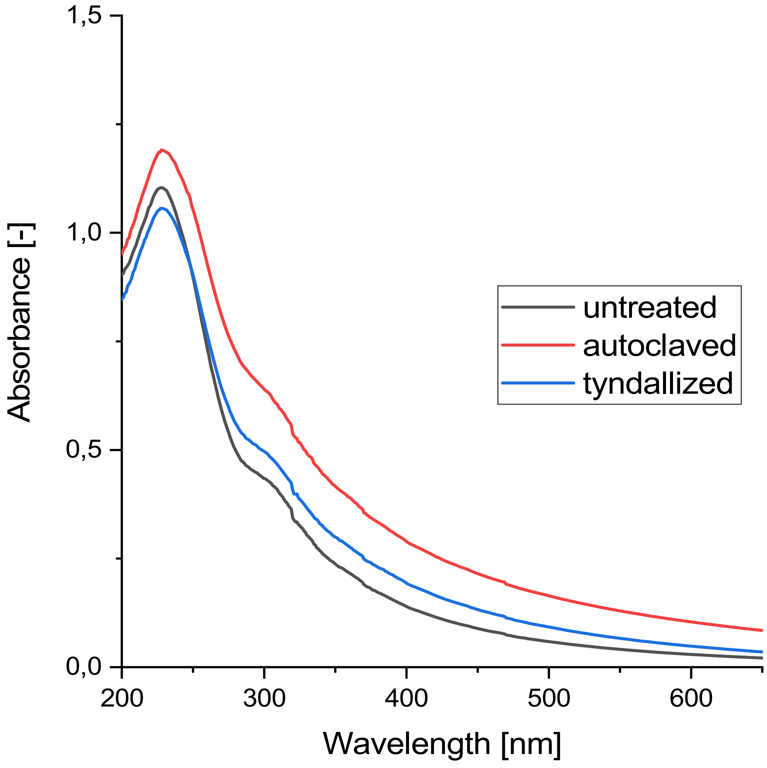

Figure S1. Comparison of the UV-vis spectra of 25 mg/L GO suspensions prior and after sterilization treatments

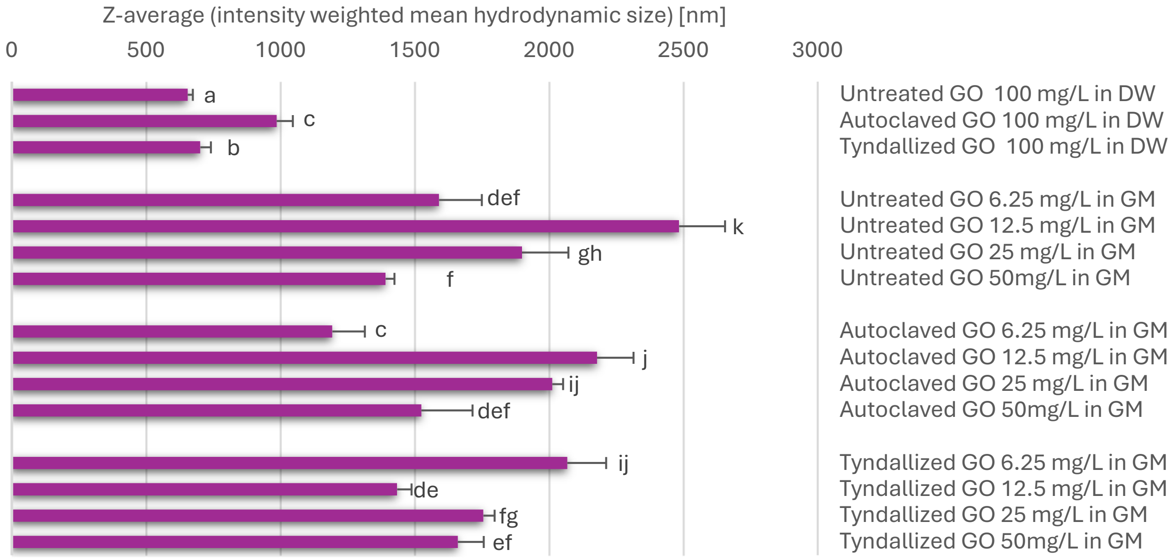

Figure S2. Z-average values measured in the GO stock suspensions and in the assembled test systems reported from n = 3 determinations per sample. (DW: distilled water; GM: growth medium)

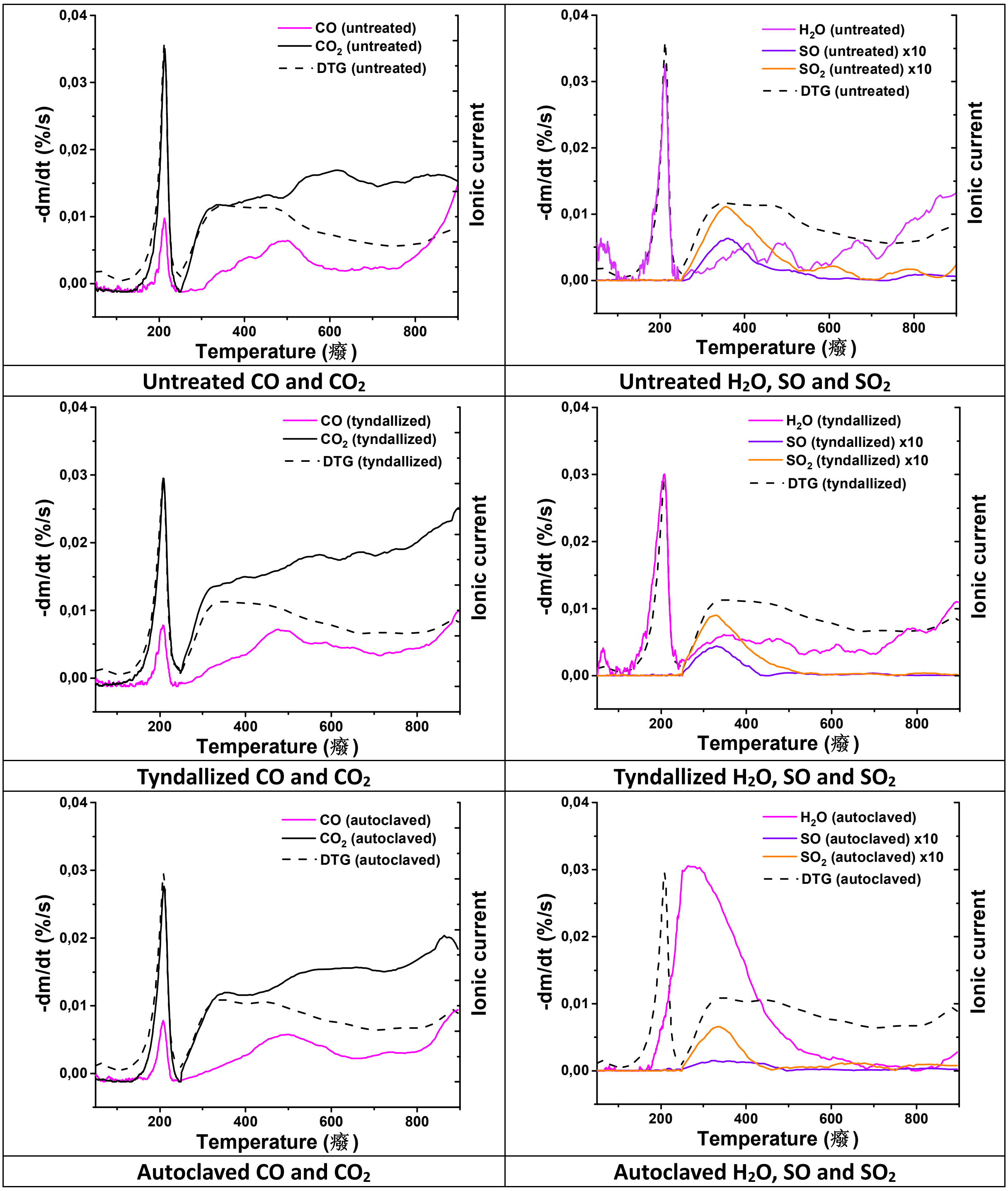

Figure S3. TG/MS analysis results of the pristine and heat-sterilized GO suspensions.

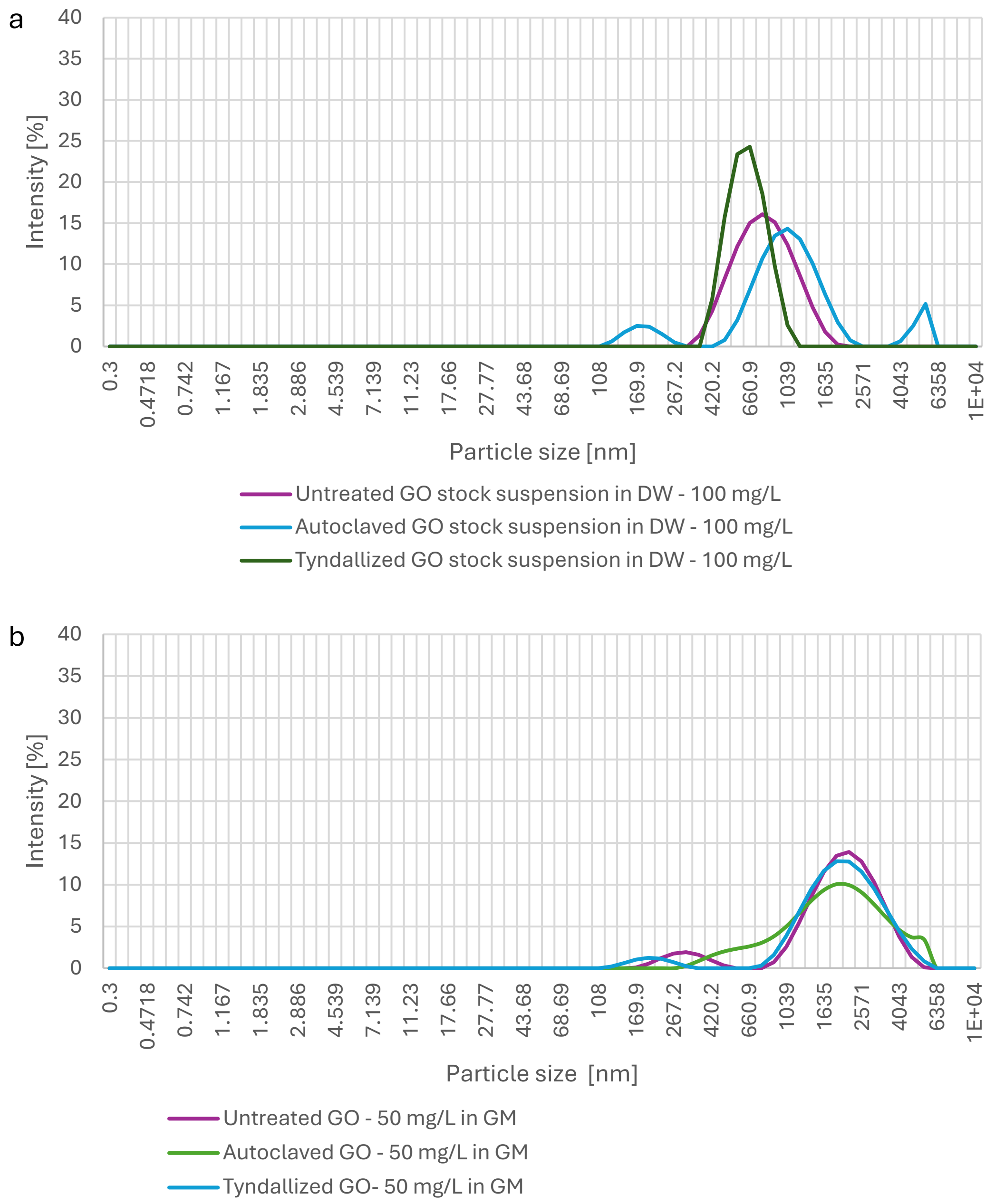

Figure S4. Particle size distribution of undiluted original GO stock suspensions (a) and of the untreated, autoclaved and tyndallized GO suspensions diluted with *Daphnia magna* growth medium (b).

Table S2a. Factorial ANOVA results to evaluate effects of sterilization methods and GO-concentration on the heart rate and feeding activity of *Daphnia magna*. Bold numbers indicate significant differences at $p < 0.05$.

Univariate Tests of Significance
Sigma Restricted Parameterization
Effective Hypothesis Decomposition

Daphnia magna heart rate 24 h				
Effect	Degr. of Freedom	Mean Square	F ratio	p value
Intercept	***1***	***31343621***	***90091.54***	***0.000000***
Sterilization	***2***	***49788***	***143.11***	***0.000000***
Concentration	***4***	***58501***	***168.15***	***0.000000***
Sterilization*concentration	***8***	***3519***	***10.11***	***0.000000***
Error	386	348		
Daphnia magna heart rate 48 h				
Effect	Degr. of Freedom	MS	F	p value
Intercept	***1***	***24888735***	***58916.00***	***0.000000***
Sterilization	***2***	***18110***	***42.87***	***0.000000***
Concentration	***4***	***49880***	***118.08***	***0.000000***
Sterilization*concentration	***8***	***1071***	***2.54***	***0.010700***
Error	369	422		
Daphnia magna feeding activity 24 h				
	Degr. of Freedom	MS	F	p value
Intercept	***1***	***620726080***	***5008.653***	***0.000000***
Sterilization	***2***	***21299430***	***171.866***	***0.000000***
Concentration	***4***	***14861914***	***119.921***	***0.000000***
Sterilization*concentration	***8***	***716693***	***5.783***	***0.000009***
Error	77	123931		
Daphnia magna feeding activity 48 h				
	Degr. of Freedom	MS	F	p value
Intercept	***1***	***606208404***	***5742.870***	***0.000000***
Sterilization	***2***	***18800132***	***178.102***	***0.000000***
Concentration	***4***	***17682861***	***167.517***	***0.000000***
Sterilization*concentration	***8***	***2086309***	***19.764***	***0.000000***
Error	89	105558		

Table S2b. Factorial ANOVA results to evaluate effects of sterilization methods and GO-concentration on the oxidative stress parameters of *Daphnia magna*. Bold numbers indicate significant differences at $p < 0.05$.

Univariate Tests of Significance
Sigma Restricted Parameterization
Effective Hypothesis Decomposition

	Degr. of Freedom	Mean Square	F ratio	p value
ROS production 24 h				
Intercept	***1***	***481197.9***	***15270.69***	***0.000000***
Sterilization	***2***	***112.9***	***3.58***	***0.047875***
Concentration	***4***	***1528.1***	***48.50***	***0.000000***
Sterilization*concentration	***8***	***199.5***	***6.33***	***0.000472***
Error	19	31.5		
GPx specific enzyme activity 24 h				
	Degr. of Freedom	MS	F	p value
Intercept	***1***	***44.40848***	***1857.330***	***0.000000***
Sterilization	***2***	***0.13102***	***5.480***	***0.010630***
Concentration	***4***	***2.68170***	***112.159***	***0.000000***
Sterilization*concentration	***8***	***0.06404***	***2.678***	***0.028274***
Error	25	0.02391		
GST specific enzyme activity 24 h				
	Degr. of Freedom	MS	F	p value
Intercept	***1***	***0.427910***	***342.6757***	***0.000000***
Sterilization	***2***	***0.019448***	***15.5745***	***0.000046***
Concentration	***4***	***0.010206***	***8.1728***	***0.000262***
Sterilization*concentration	8	0.002786	2.2309	0.061464
Error	24	0.001249		

Table S2c. Factorial ANOVA results to evaluate effects of sterilization methods and GO-concentration on the effective concentration values of the different *Daphnia magna* ecotoxicity endpoints. Bold numbers indicate significant differences at $p < 0.05$.

Univariate Tests of Significance
Sigma Restricted Parameterization
Effective Hypothesis Decomposition

EC_{20} Feeding activity				
	Degr. of Freedom	Mean Square	F ratio	p value
Intercept	***1***	***610.0525***	***17120.26***	***0.000000***
Sterilization	***1***	***97.5805***	***2738.46***	***0.000000***
Concentration	***2***	***179.2114***	***5029.31***	***0.000000***
Sterilization*concentration	***2***	***78.5389***	***2204.08***	***0.000000***
Error	12	0.0356		
EC_{50} Feeding activity				
	Degr. of Freedom	MS	F	p value
Intercept	***1***	***8252.270***	***7901.231***	***0.000000***
Sterilization	***1***	***9.031***	***8.647***	***0.012358***
Concentration	***2***	***2037.412***	***1950.743***	***0.000000***
Sterilization*concentration	2	1.340	1.283	0.312678
Error	12	1.044		
EC_{20} Heart rate				
	Degr. of Freedom	MS	F	p value
Intercept	***1***	***5901.325***	***4743.480***	***0.000000***
Sterilization	***1***	***1711.125***	***1375.401***	***0.000000***
Concentration	***2***	***61.299***	***49.272***	***0.000002***
Sterilization*concentration	***2***	***351.094***	***282.209***	***0.000000***
Error	12	1.244		
